# Supplementary material for: Effects of strength training on repeated sprint ability in team sports players: a systematic review
Source: PeerJ. 2024 Aug 6;12:e17756. doi: 10.7717/peerj.17756 (PMC11313415; doi:10.7717/peerj.17756)
Supplement: Supplemental Information 1 [file peerj-12-17756-s001.docx]

**Table S1.** 1 repetition maximum test

| **Author** | **1 RM test** | **Group** | **N° of Subjects** | **Pre-test (mean ± SD)** | **Post-test (mean ± SD)** | **ES**  **intra-group** | **p-value**  **intra-group** | **ES between-group** | **p-value between-group (between EG and CG)** | **ES (group × time)** | **p-value (group × time)** | **% Δ** |
| --- | --- | --- | --- | --- | --- | --- | --- | --- | --- | --- | --- | --- |
| **Chatzinikolaou et al. (2018)** | 1 RM full squat (kg) | STG | 12 | No reported |  |  | < 0.05* |  | < 0.05* |  |  | 21.1 |
|  |  | CG | 10 | No reported |  |  | < 0.05* |  |  |  |  | -7.9 |
| **Durán-Custodio et al. (2023)** | Not assessed # | STG | 10 |  |  |  |  |  |  |  |  |  |
|  |  | CG | 10 |  |  |  |  |  |  |  |  |  |
| **Gonzalo-Skok et al. (2016)** | Incremental Load Test in Leg Press of Pmax (w) ## | RPMG | 11 | 937.4 ± 248.4 |  |  |  |  |  |  |  |  |
|  |  | CG | 11 | Not assessed |  |  |  |  |  |  |  |  |
| **Hammami et al. (2019)** | 1 RM half squat (kg) | CCTG | 14 | 73.7 ± 8.0 | 91.1 ± 7.3 |  | < 0.05* |  |  | 0.17 | 0.003* | 23.6 |
|  |  | CG | 14 | 76.0 ± 7.4 | 80.2 ± 7.5 |  | > 0.05 |  |  |  |  | 5.53 |
| **Hammami et al. (2018)** | 1 RM half squat (kg) | STG | 19 | 99.8 ± 7.5 | 125.1 ± 4.7 |  | ≤ 0.01* |  |  | 0.156 | 0.002* | 25.4 |
|  |  | CG | 12 | 94.3 ± 13.9 | 100.1 ± 19.0 |  | > 0.05 |  |  |  |  | 6.2 |
| **Hammami et al. (2017b)** | 1 RM half squat (kg) | CCTG | 17 | 95 ± 10 | 136 ± 8 |  | ≤ 0.001* |  |  | 0.333 | 0.000* | 43.2 |
|  |  | CG | 12 | 94 ± 14 | 99 ± 19 |  | > 0.05 |  |  |  |  | 5.3 |
| **Hammami et al. (2017a)** | 1 RM half squat (kg) | CCTG | 16 | 96.3 ± 9.7 | 135.8 ± 8.4 |  |  |  | ≤ 0.001* | 0.316 | 0.000* | 41.02 |
|  |  | STG | 16 | 101.1 ± 7.6 | 125.3 ± 4.9 |  |  |  | ≤ 0.001* |  |  | 23.9 |
|  |  | CG | 12 | 95.6 ± 12.1 | 101.4 ± 17.3 |  |  |  |  |  |  | 6.07 |
| **Hermassi et al. (2017)** | 1 RM half squat (kg) | STG | 12 | 206 ± 12.5 | 236 ± 9.50 | 2.68 |  |  |  | η2 0.740 | < 0.001* | 14.6 |
|  |  | CG | 10 | 205 ± 17.2 | 202 ± 13.2 | 0.20 |  |  |  |  |  | -1.5 |
| **Torres et al. (2017)** | 1 RM estimated by Isoinertial full squat loading test (kg) | STG | 12 | 88.6 ± 13.8 | 102.8 ± 10.1 | 0.87 | < 0.001* |  | < 0.01* |  | < 0.001* | 16.03  -0.97 |
|  |  | CG | 10 | 92.5 ± 20.7 | 91.6 ± 17.9 | −0.01 | > 0.05 |  |  |  |  |  |
| **Torres et al. (2018)** | 1 RM estimated by Isoinertial full squat loading test (kg) | STG | 12 | 88.6 ± 13.8 | 102.8 ± 10.1 | 0.87 | < 0.001* | 0.79 | 0.002* |  | < 0.001* | 16.03 |
|  |  | CG | 10 | 92.5 ± 20.7 | 91.6 ± 17.9 | − 0.01 | > 0.05 |  |  |  |  | -0.97 |

| **Abbreviations. CG:** control group **RMPG:** repeated maximal power group **STG:** strength training group **CCTG:** complex contrast training group **1 RM:** repetition maximum  ***:** 1 RM test presented significant change p ≤ 0.05  **#:** Not assessed pre and post-test, 1 RM was calculated as follows: (1 RM = Weight lifted in kilograms × [1 + 0.025 × n repetitions]) and the corresponding percentages were calculated individually for each player and exercise  ##: only evaluated pre-test in RPMG |
| --- |
