# Supplementary material for: Effects of strength training on repeated sprint ability in team sports players: a systematic review
Source: PeerJ. 2024 Aug 6;12:e17756. doi: 10.7717/peerj.17756 (PMC11313415; doi:10.7717/peerj.17756)
Supplement: Supplemental Information 3 [file peerj-12-17756-s003.docx]

| **Section and Topic** | **Item #** | **Checklist item** | **Location where item is reported** |
| --- | --- | --- | --- |
| **TITLE** | | | **Line 1** |
| Title | 1 | Identify the report as a systematic review. | Effects of strength training on repeated sprint ability in team sports players: A Systematic review **Line 2** |
| **ABSTRACT** | | | **Line 28-51** |
| Abstract | 2 | See the PRISMA 2020 for Abstracts checklist. |  |
| **INTRODUCTION** | | | **Line 86-156** |
| Rationale | 3 | Describe the rationale for the review in the context of existing knowledge. | Further, no previous studies have systematically explored the effects of strength training (characterized by actions against external resistance) and complex training on RSA in different team sports **Line 150-152** |
| Objectives | 4 | Provide an explicit statement of the objective(s) or question(s) the review addresses. | Therefore, this systematic review aims to analyze the existing evidence on the effects of strength and complex training on RSA in team sports players compared to a control group **Line 152-156** |
| **METHODS** | | | **Line 158-229** |
| Eligibility criteria | 5 | Specify the inclusion and exclusion criteria for the review and how studies were grouped for the syntheses. | Inclusion and exclusion criteria were structured through a PICOS approach (participants, intervention, comparators, outcomes, and study design) and are presented in Table 1. **Line 169-185** |
| Information sources | 6 | Specify all databases, registers, websites, organisations, reference lists and other sources searched or consulted to identify studies. Specify the date when each source was last searched or consulted. | The PubMed, Web of Science (Core Collection), and Scopus databases were considered until August 27, 2023 **Line 164-165**  In addition, manual searches were performed by consulting grey literature, and reference lists were analyzed from selected articles in search of possible  Studies **Line 199-200** |
| Search strategy | 7 | Present the full search strategies for all databases, registers and websites, including any filters and limits used. | The supplementary material (Table S1) shows the search strategies used in the three databases **Line 165-166**  Search criteria were performed using the following descriptors: “ballistic training,” “resistance training” [Mesh],” strength training” [Mesh], “complex training,” “contrast training,” “weight training,” “repeated change of direction,” “repeated sprint ability,” “repeated sprint exercise.” **Line 191-199** |
| Selection process | 8 | Specify the methods used to decide whether a study met the inclusion criteria of the review, including how many reviewers screened each record and each report retrieved, whether they worked independently, and if applicable, details of automation tools used in the process. | Articles were selected by title and abstract; then, the full text were reviewed to finally apply the inclusion criteria described in Table 1. Duplicate articles were removed **Line 203-207** |
| Data collection process | 9 | Specify the methods used to collect data from reports, including how many reviewers collected data from each report, whether they worked independently, any processes for obtaining or confirming data from study investigators, and if applicable, details of automation tools used in the process. | Data extraction was performed by the lead author using a standardized form created in Microsoft Excel **Line 210-211** |
| Data items | 10a | List and define all outcomes for which data were sought. Specify whether all results that were compatible with each outcome domain in each study were sought (e.g. for all measures, time points, analyses), and if not, the methods used to decide which results to collect. | The repeated sprint ability was considered the primary variable, measured with different RSA tests recorded in execution time in seconds as mean ± standard deviation (SD) **Line 211-212**  From the extracted data, means, SD, first author’s last name, year of publication of the studies, sport, competition season, training intervention characteristics **Line 212-216** |
|  | 10b | List and define all other variables for which data were sought (e.g. participant and intervention characteristics, funding sources). Describe any assumptions made about any missing or unclear information. | and descriptive characteristics of team sports players [number of subjects per group (size), chronological age (years) **Line 217-219** |
| Study risk of bias assessment | 11 | Specify the methods used to assess risk of bias in the included studies, including details of the tool(s) used, how many reviewers assessed each study and whether they worked independently, and if applicable, details of automation tools used in the process. | The Physiotherapy Evidence Database (PEDro) scale was used to assess the risk of bias and methodological quality of the included studies **Line 222-229** |
| Effect measures | 12 | Specify for each outcome the effect measure(s) (e.g. risk ratio, mean difference) used in the synthesis or presentation of results. | The repeated sprint ability was considered the primary variable, measured with different RSA tests recorded in execution time in seconds as mean ± standard deviation (SD) **Line 211-212** |
| Synthesis methods | 13a | Describe the processes used to decide which studies were eligible for each synthesis (e.g. tabulating the study intervention characteristics and comparing against the planned groups for each synthesis (item #5)). | Not applicable |
|  | 13b | Describe any methods required to prepare the data for presentation or synthesis, such as handling of missing summary statistics, or data conversions. | Not applicable |
|  | 13c | Describe any methods used to tabulate or visually display results of individual studies and syntheses. | Not applicable |
|  | 13d | Describe any methods used to synthesize results and provide a rationale for the choice(s). If meta-analysis was performed, describe the model(s), method(s) to identify the presence and extent of statistical heterogeneity, and software package(s) used. | Not applicable |
|  | 13e | Describe any methods used to explore possible causes of heterogeneity among study results (e.g. subgroup analysis, meta-regression). | Not applicable |
|  | 13f | Describe any sensitivity analyses conducted to assess robustness of the synthesized results. | Not applicable |
| Reporting bias assessment | 14 | Describe any methods used to assess risk of bias due to missing results in a synthesis (arising from reporting biases). | Not applicable |
| Certainty assessment | 15 | Describe any methods used to assess certainty (or confidence) in the body of evidence for an outcome. | No |
| **RESULTS** | | | **Line 264-395** |
| Study selection | 16a | Describe the results of the search and selection process, from the number of records identified in the search to the number of studies included in the review, ideally using a flow diagram. | Through the realization of the literature search in the selected databases, a total of 678 studies were identified in PubMed, 832 in Web of Science, and 1568 from Scopus. After removing duplicates, meta-analyses, systematic reviews, and abstracts, a total of 2544 results of full texts 270 were available **Line 267-273** |
|  | 16b | Cite studies that might appear to meet the inclusion criteria, but which were excluded, and explain why they were excluded. | The full texts of the 36 articles were read using the inclusion and exclusion criteria specified for the eligibility of the studies. Finally, nine articles were selected (Figure 1) **Line 271-273** and the full-text articles excluded were recorded in a Microsoft Excel table with the causes for their exclusion **Line 204-205** |
| Study characteristics | 17 | Cite each included study and present its characteristics. | Among the main characteristics of nine studies (Chatzinikolaou et al., 2018; Gonzalo-Skok et al., 2016; Hammami et al., 2019; **Line 276-353** |
| Risk of bias in studies | 18 | Present assessments of risk of bias for each included study. | The methodological quality of the 9 studies included in this systematic review was quantified through the PEDro scale, yielding moderate quality in the 9 studies (5 to 6 points) **Line 393-395** |
| Results of individual studies | 19 | For all outcomes, present, for each study: (a) summary statistics for each group (where appropriate) and (b) an effect estimate and its precision (e.g. confidence/credible interval), ideally using structured tables or plots. | EGs with junior male soccer players had no significant changes intergroup (all trivial ES; <0.2) **Line 355-383** |
| Results of syntheses | 20a | For each synthesis, briefly summarise the characteristics and risk of bias among contributing studies. | Not applicable |
|  | 20b | Present results of all statistical syntheses conducted. If meta-analysis was done, present for each the summary estimate and its precision (e.g. confidence/credible interval) and measures of statistical heterogeneity. If comparing groups, describe the direction of the effect. | Not applicable |
|  | 20c | Present results of all investigations of possible causes of heterogeneity among study results. | Not applicable |
|  | 20d | Present results of all sensitivity analyses conducted to assess the robustness of the synthesized results. | Not applicable |
| Reporting biases | 21 | Present assessments of risk of bias due to missing results (arising from reporting biases) for each synthesis assessed. | Not applicable |
| Certainty of evidence | 22 | Present assessments of certainty (or confidence) in the body of evidence for each outcome assessed. | No |
| **DISCUSSION** | | | **Line 412-571** |
| Discussion | 23a | Provide a general interpretation of the results in the context of other evidence. | Three studies (Gonzalo-Skok et al., 2016; Hermassi et al., 2017; Torres-Torrelo et al., 2018) presented significant changes in their EG in specific measures of RSA (best Time and Mean Time). These results are coincident with Sanchez-Sanchez et al. (2022) **Line 420-426** |
|  | 23b | Discuss any limitations of the evidence included in the review. | Firstly, since allocation was concealed, the moderate quality of the studies could produce systematic biases in random allocation **Line 561-562** |
|  | 23c | Discuss any limitations of the review processes used. | The small number of studies on the effectiveness of strength training on RSA does not allow us to compare the results regarding the characteristics of the subjects and intervention of the training **Line 563-565** |
|  | 23d | Discuss implications of the results for practice, policy, and future research. | According to the findings of the articles reviewed in the present study, coaches should consider strength and complex training to improve RSA in team sports **Line 588-594**  Future studies should include ballistic exercises where higher RFDs can be achieved and exercises in the horizontal direction **Line 568-569** |
| **OTHER INFORMATION** | | |  |
| Registration and protocol | 24a | Provide registration information for the review, including register name and registration number, or state that the review was not registered. | No |
|  | 24b | Indicate where the review protocol can be accessed, or state that a protocol was not prepared. | No |
|  | 24c | Describe and explain any amendments to information provided at registration or in the protocol. | No |
| Support | 25 | Describe sources of financial or non-financial support for the review, and the role of the funders or sponsors in the review. | This research did not receive external funding **Line 600** |
| Competing interests | 26 | Declare any competing interests of review authors. | The authors declare no conflict of interest **Line 602** |
| Availability of data, code and other materials | 27 | Report which of the following are publicly available and where they can be found: template data collection forms; data extracted from included studies; data used for all analyses; analytic code; any other materials used in the review. | No |

*From:*  Page MJ, McKenzie JE, Bossuyt PM, Boutron I, Hoffmann TC, Mulrow CD, et al. The PRISMA 2020 statement: an updated guideline for reporting systematic reviews. BMJ 2021;372:n71. doi: 10.1136/bmj.n71

For more information, visit: <http://www.prisma-statement.org/>
