# Supplementary material for: Effects of strength training on repeated sprint ability in team sports players: a systematic review
Source: PeerJ. 2024 Aug 6;12:e17756. doi: 10.7717/peerj.17756 (PMC11313415; doi:10.7717/peerj.17756)
Supplement: Supplemental Information 4 [file peerj-12-17756-s004.docx]

**Rationale for Conducting the Systematic Review**

**Dear Editor:**

In the last decade, a considerable amount of published studies as meta-analyses have analyzed the effects of strength training (characterized by actions against external resistance) and of complex/contrast training of the lower extremity with substantial improvements in the sprint performance in team sports players (Seitz et al., 2014; Freitas et al., 2017; Thapa et al., 2020). The improvements are likely due to the strong relationship that exists between maximal muscular strength with the rate of force development (RFD) and external mechanical power, which can translate in practice into shorter ground contact times and improver sprint performance in short distances (Suchomel, Nimphius & Stone, 2016).

On the other hand, the effects of strength and complex/contrast training on repeated sprint ability (RSA) have been less addressed in the literature because, unlike a single sprint, RSA requires a complex interaction both neuro-mechanical properties and high anaerobic power for maintain or improving initial sprint performance and physiological/metabolic factors for maintain or improving the recovery between sprints (Girard, Mendez-Villanueva & Bishop, 2011), which represents a challenge for improving both factors simultaneously using only strength or complex/contrast training. For example, so that lower-body strength transfers positively to sprint performance, maximal or explosive/power strength training requires performing sets with few repetitions, long recovery periods between sets (2-3 min), and low level of neural fatigue to develop high levels of RFD and/or power (Seitz et al., 2014).

According to the authors' knowledge, only one meta-analysis study has explored the effects of complex/contrast training on RSA in male soccer players; however, some studies did not include control groups, so the efficacy is not adequately addressed (Thapa et al., 2022). Those results could lead to biases and should not be extrapolated with male or female athletes of other team sports different from soccer. Further, they were analyzed without any details regarding the intervention protocols (Thapa et al., 2022). We also do not find studies of systematic review or meta-analysis using strength or complex/contrast training on RSA in different team sports, which allows knowing the effects in sports with different physical and technical demands. The above represents a gap in the literature and an excellent opportunity for resolving those questions through a systematic review, and it is the orderly and explicit evaluation of the literature from a straightforward research question along with a critical analysis of the information. Therefore, it allows us to obtain answers based on grouped results that provide a global vision of the problem to solve and not on isolated results of individual studies. Therefore, this systematic review aims to analyze the existing evidence on the effects of strength and complex training on RSA in team sports players compared to a control group.

**The contribution that it makes to knowledge in light of previously published related reports, including other meta-analyses and systematic reviews**

This systematic review will allow us to address the strengths and weaknesses of the methodology, the intervention protocols used, and the risk of bias and methodological quality of the included studies, among other factors. For example, none of the studies reported whether the allocation was concealed, which could produce systematic biases in random allocation.

Regarding the intervention protocols, they were not strength exercises of a ballistic nature or weightlifting derivatives, which could generate a greater RFD, nor specific exercises to produce force in the anterior-posterior direction that place greater specificity on the knee and hip extensor muscles that are fundamental to effectively apply forward-oriented ground reaction force during la acceleration of every sprint (Junge et al., 2021).

The understanding of the results of this systematic review (best time, mean time, fatigue index, and total time) regarding the multiple factors that interact during repeated sprinting, along with the critical analysis of the intervention protocols, will contribute to studies future to develop training programs more specific and efficient according to the technical demands required by the RSA.

**References**

Freitas TT, Martinez-Rodriguez A, Calleja-González J, Alcaraz PE. 2017. Short-term adaptations following Complex Training in team-sports: A meta-analysis. *PloS one* 12:e0180223. DOI: 10.1371/journal.pone.0180223.

Girard O, Mendez-Villanueva A, Bishop D. 2011. Repeated-sprint ability - part I: factors contributing to fatigue. *Sports medicine (Auckland, N.Z.)* 41:673–694. DOI: 10.2165/11590550-000000000-00000.

Junge N, Lundsgaard A, Hansen MF, Samozino P, Morin J-B, Aagaard P, Contreras B, Nybo L. 2021. Force-velocity-power profiling of maximal effort sprinting, jumping and hip thrusting: Exploring the importance of force orientation specificity for assessing neuromuscular function. *Journal of Sports Sciences* 39:2115–2122. DOI: 10.1080/02640414.2021.1920128.

Seitz LB, Reyes A, Tran TT, de Villarreal ES, Haff GG. 2014. Increases in Lower-Body Strength Transfer Positively to Sprint Performance: A Systematic Review with Meta-Analysis. *Sports Medicine* 44:1693–1702. DOI: 10.1007/s40279-014-0227-1.

Suchomel TJ, Nimphius S, Stone MH. 2016. The Importance of Muscular Strength in Athletic Performance. *Sports medicine (Auckland, N.Z.)* 46:1419–1449. DOI: 10.1007/s40279-016-0486-0.

Thapa RK, Lum D, Moran J, Ramirez-Campillo R. 2020. Effects of Complex Training on Sprint, Jump, and Change of Direction Ability of Soccer Players: A Systematic Review and Meta-Analysis. *Frontiers in psychology* 11:627869. DOI: 10.3389/fpsyg.2020.627869.

Thapa R, Narvariya P, Weldon A, Talukdar K, Ramirez-Campillo R. 2022. Can complex contrast training interventions improve soccer players' aerobic endurance, maximal strength, and repeated sprint ability? A systematic review and meta-analysis. *Montenegrin Journal of Sports Science and Medicine* 11. DOI: 10.26773/mjssm.220906.
